# Improving the Substrate Affinity and Catalytic Efficiency of β-Glucosidase Bgl3A from *Talaromyces leycettanus* JCM12802 by Rational Design

**DOI:** 10.3390/biom11121882

**Published:** 2021-12-15

**Authors:** Wei Xia, Yingguo Bai, Pengjun Shi

**Affiliations:** 1 Institute of Food Science and Technology, Chinese Academy of Agricultural Sciences, Beijing 100081, China; weixia@jiangnan.edu.cn; 2 Institute of Animal Science, Chinese Academy of Agricultural Sciences, Beijing 100193, China

**Keywords:** β-glucosidase, cellobiose, enzyme engineering, substrate affinity, molecular dynamics simulation

## Abstract

Improving the substrate affinity and catalytic efficiency of β-glucosidase is necessary for better performance in the enzymatic saccharification of cellulosic biomass because of its ability to prevent cellobiose inhibition on cellulases. Bgl3A from *Talaromyces leycettanus* JCM12802, identified in our previous work, was considered a suitable candidate enzyme for efficient cellulose saccharification with higher catalytic efficiency on the natural substrate cellobiose compared with other β-glucosidase but showed insufficient substrate affinity. In this work, hydrophobic stacking interaction and hydrogen-bonding networks in the active center of Bgl3A were analyzed and rationally designed to strengthen substrate binding. Three vital residues, Met36, Phe66, and Glu168, which were supposed to influence substrate binding by stabilizing adjacent binding site, were chosen for mutagenesis. The results indicated that strengthening the hydrophobic interaction between stacking aromatic residue and the substrate, and stabilizing the hydrogen-bonding networks in the binding pocket could contribute to the stabilized substrate combination. Four dominant mutants, M36E, M36N, F66Y, and E168Q with significantly lower *K*_m_ values and 1.4–2.3-fold catalytic efficiencies, were obtained. These findings may provide a valuable reference for the design of other β-glucosidases and even glycoside hydrolases.

## 1. Introduction

Cellulose is the most abundant polysaccharide in nature and constitutes the highest proportion of municipal and agricultural wastes. Additionally, it represents the most valuable source of renewable energy and raw materials [1]. The worldwide consensus is that the efficient utilization of cellulosic agricultural wastes is critical in generating sustainable biofuel production methods [2]. The enzymatic degradation of cellulose to glucose is generally accomplished by a synergic action of three kinds of glycoside hydrolases, such as endo-β-glucanase (EG, EC 3.2.1.4), cellobiohydrolase (CBHs, EC 3.2.1.91), and β-glucosidase (BGLs, EC 3.2.1.21) [3,4]. Fungi, as the primary producers of cellulose-degrading enzymes, have received most of the attention regarding biotechnological applications. However, as previously reported, the lack of endogenous β-glucosidase is always the fatal defect of current industrial microorganisms used for the enzymatic degradation of cellulose, such as *Trichoderma reesei* [5,6]. This is because the end-product inhibition of CBHs by cellobiose, which is the natural substrate of β-glucosidase, can seriously reduce the overall conversion rate of cellulose into glucose [7,8]. Therefore, β-glucosidases with high qualities are essential for enhancing the utilization efficiency of cellulosic agricultural wastes [9,10].

β-glucosidases, mainly catalyzing the hydrolysis of the β-1,4-glycosidic linkage in various disaccharides, oligosaccharides, and alkyl- and aryl-β-d-glucosides, have been classified into GH families 1, 3, 5, 9, 30, and 116 based on their amino acid sequences [11,12,13]. The different β-glucosidases vary significantly regarding enzymatic properties, such as reaction optimums, substrate specificities, catalytic efficiencies, tolerance to unfavorable conditions, and inhibition constants for glucose [8]. Most of the microbial β-glucosidases employed today in cellulose hydrolysis belong to GH family 3 [11,14]. Increasing research has focused on the protein structure, ligand-binding mode, and critical residues of GH3 β-glucosidases. Structural analysis has revealed three conserved substrate recognizing residues for the subsite +1 of cellobiose in the β-glucosidases capable of hydrolyzing cellobiose, for instance, Trp68, Phe305, and Tyr511 of AaBGL1 from *Aspergillus aculeatus* (PDB 4IIB) [15], and Trp37, Phe260, and Tyr443 of HjCel3A from *Hypocrea jecorina* (PDB 3ZYZ) [16]. Moreover, several recent studies have reported that several critical residues at the entrance or inside the catalytic pocket could influence the enzymatic properties of β-glucosidases. These include the Ile substitution of conserved Trp at the pocket entrance that makes HiBgl3B from *Humicola insolens* Y1 be a strict aryl-β-glucosidase [11]. Furthermore, three amino acid changes contribute markedly to the thermostability of β-glucosidase BglC from *Thermobifida fusca* [17], and R97 and Y331 could modulate the optimum pH of GH1 β-glycosidase from *Spodoptera frugiperda* [18].

Significant efforts have been made to improve the enzymatic properties of the existing GH3 enzymes owing to the practical benefit of β-glucosidases in the biofuel industry. A series of valuable promotions have been achieved by newly developed structure-based rational design, which was assisted by computational algorithms in the enzyme engineering of GH3 β-glucosidases [19]. Beneficially combined mutants with increased hydrolytic activity for synthetic and natural substrates have been obtained by directed evolution of a fungal GH3 β-glucosidase BGL1 in *Saccharomyces cerevisiae* [20]. The Q201E mutant of AaBGL1, generated by site-saturation mutagenesis, was found to have 2.7-times higher *k*_cat_/*K*_m_ toward cellobiose than the WT enzyme [21]. Substitution of Trp512 in β-glucosidase from *Clavibacter michiganensis* has been shown to transform the regioselectivity for hydrolyzing gypenoside XVII [22]. Furthermore, the catalytic efficiency in quercetin-4’-glucoside hydrolysis of *Thermotoga maritima* β-glucosidase A was enhanced by site-directed mutagenesis [23]. However, most studies were conducted using *p*NPG as the representative substrate molecule, and few modifications were made on the substrate affinity and catalytic efficiency of GH3 β-glucosidases toward their natural substrate cellobiose. To improve the practical economic benefits of the enzymatic saccharifications of cellulosic biomass and the subsequent bioethanol production, more attention must be paid to the catalytic performances of β-glucosidases on cellobiose [9]. Therefore, developing β-glucosidases with reasonable substrate specificity and high catalytic efficiency is of great importance for the biotransformation of rare ginsenosides.

Our previous work identified and characterized the highly efficient GH3 β-glucosidase (Bgl3A) from *Talaromyces leycettanus* JCM12802. This had excellent application prospects with a relatively high specific activity and catalytic efficiency of 905 U/mg and 9096/s/mM on *p*NPG, respectively [14]. Its high catalytic efficiency benefited from the high *k*_cat_ value. However, the specific activity and catalytic efficiency of Bgl3A on cellobiose were much lower (265.5 U/mg and 75.8/s/mM) because of its low affinity towards cellobiose. The K_m_ value of Bgl3A on cellobiose was 10.4 mM, a 57-fold higher value compared with *p*NPG. It indicated that, although the *k*_cat_/*K*_m_ value of 75.8/s/mM was at a relatively high level among GH3 β-glucosidases, Bgl3A could not work well when the concentration of cellobiose was not very high. Therefore, the low affinity towards cellobiose was a significant bottleneck for practical use of Bgl3A in the biofuel industry, considering that cellobiose was the primary natural substrate of β-glucosidases in the process of cellulose saccharification. The purpose of this study was to improve the substrate affinity of Bgl3A towards cellobiose by enzyme engineering. Using the site-directed saturation mutagenesis method, two improved mutants with 2.3-fold higher catalytic efficiencies on cellobiose were obtained.

## 2. Materials and Methods

### 2.1. Strains, Plasmids, and Materials

Recombinant plasmid pPIC9 harboring the coding sequence *bgl*3A (gene bank accession KU363626), constructed in our previous work [14], was used as the template for site-saturation mutagenesis. *Pichia pastoris* strain GS115 (Invitrogen, Carlsbad, CA, USA) was employed as a heterologous expression host for protein preparation. The substrates 4-nitrophenyl β-d-glucopyranoside (*p*NPG), cellobiose, gentibiose, and salicin were purchased from Sigma–Aldrich (St. Louis, MO, USA). *LA Taq* DNA polymerase, restriction endonucleases, and DNA purification kit were purchased from TaKaRa (Otsu, Japan). DNA ligase and total RNA isolation system kit were purchased from Promega (Madison, WI, USA). All other chemicals were of analytic grade and commercially available.

### 2.2. Sequence Analysis, Homology Modeling, and Docking

The multiple sequence alignment of Bgl3A and other typical GH3 β-glucosidases was performed using ClustalX version 2.1, followed by a rendering of sequence similarities and secondary structure information in the aligned sequences by the online tool ESPript (http://espript.ibcp.fr/ESPript/ESPript/) (accessed on 3 October 2021). The evolutionary conservation analysis was performed using the Weblogo program (http://weblogo.berkeley.edu/logo.cgi) (accessed on 9 December 2021). The homology model of the three-dimensional structure of wild-type Bgl3A was homology modeled by the Swiss Model server (https://www.swissmodel.expasy.org/) (accessed on 9 December 2021) with the crystal structure of *Hj*Cel3A from *H. jecorina* (PDB: 3ZYZ; with 73% identity) as the template. The obtained optimal model of Bgl3A was evaluated using Verify3D and PROCHECK programs (Supplemental Figure S1). The pairwise cation-π interactions were predicted using the online tool PIC (Protein Interactions Calculator, http://pic.mbu.iisc.ernet.in/job.html) (accessed on 9 December 2021), and the evaluation standard required that the distance between the basic and aromatic amino acid was within 6 Å and the angle was appropriate [24].

The structure file of the ligand cellobiose, a representative of the disaccharide substrate of β-glucosidase, was generated and energy-minimized using the ChemBioOffice software (Version 14.0, CambridgeSoft Corporation, Cambridge, MA, USA). The docking of modeled Bgl3A and cellobiose was carried out by the Autodock4.2 program following the user guide. The docking grids were set as 40 × 40 × 40 with a grid spacing of 1 Å and centered on the β-carbon atom of the catalyst Glu234. The exhaustiveness for the docking poses was 50. Finally, a suitable enzyme-substrate complex Bgl3A-cellobiose was obtained using binding energy values as the evaluation criterion. The complex structure was further minimized and optimized with a 500-step molecular dynamics (MD) simulation using the Amber 18 package. To avoid the possible errors, we did energy minimization for the model before docking and MD simulation, respectively. Consequently, the binding mode of the substrate cellobiose in the obtained docking model is close to that in the template structure 3ZYZ, indicating that the docking results are reasonable to some extent. Visualization, analysis, and figure preparation of the protein and complex structures were performed with PyMOL (version 1.8.x, Delano Scientific LLC, Berkeley, CA, USA).

### 2.3. Identification of Mutagenesis Sites and Mutant Construction

Based on the structure and docking analysis, three residues (Trp35, Arg65, and Arg167) were proposed to be of great importance for binding the glycoside substrates. However, these binding residues were almost absolutely conservative in GH3 β-glucosidases. Therefore, three adjacent residues (Met36, Phe66, and Gln168), which showed less conservation and interacted with Trp35, Arg65, and Arg167, respectively, were chosen for mutagenesis to improve the catalytic properties of Bgl3A. Overlap PCR was performed to obtain gene fragments of mutated coding sequences using specific primers shown in Supplemental Table S1. The PCR products and pPIC9 vector were both digested by *EcoR* I and *Not* I and ligated by T4 ligase to construct the expression plasmids. Sequencing was carried out to verify the coding genes and successful recombination into the pPIC9 vector. The Vector NTI 11.5 software (Invitrogen, Carlsbad, CA, USA) was used to analyze the sequencing results of mutagenesis.

### 2.4. Enzyme Expression and Purification

All recombinant plasmids were digested by the single restriction enzyme *Bgl* II and the completely linearized product was transformed into *P. pastoris* GS115 competent cells by electroporation using a Gene Pulser X cell Electroporation System (Bio-Rad, Hercules, CA, USA). The cells were coated onto minimal dextrose medium plates and cultured at 30 °C for 2 days. Then, the screening of transformants with the highest enzyme activity was performed in a 10 mL tube using the method described in our previous work [14]. For shake-flask fermentation, the recombinant strains were activated in the YPD medium, followed by 2 days of propagation culture in BMGY medium (400 mL), and 2 days of induction culture in BMMY medium (200 mL) containing 1% methanol at 30 °C. The cultures were centrifuged (12,000× *g*, 4 °C, and 10 min), and the supernatant was collected and concentrated by a 5 kDa cutoff tangential flow Vivaflow ultrafiltration membrane (Vivascience, Hannover, Germany). Then, the concentrated crude enzymes were desalted by dialysis and purified using anion exchange chromatography (HiTrapQ Sepharose XL, 5 mL column, 20 mM Tris-HCl buffer, pH 8.0). Purified enzymes were subjected to sodium dodecyl sulfate-polyacrylamide gel electrophoresis (SDS-PAGE). Lastly, protein concentration measurements were obtained using the Bradford method with bovine serine albumin (BSA) as the standard.

### 2.5. Enzymatic Assays and Kinetic Parameters

The optimum temperature and pH of wild-type Bgl3A and all the mutants were determined using *p*NPG as a substrate. One unit of β-glucosidase activity was defined as the amount of enzyme that released 1 μmol of glucose per minute. For the substrate *p*NPG, the standard reaction system, containing the appropriately diluted enzyme (250 μL) and Na_2_HPO_4_-citric acid buffer of different pH values containing 2 mM *p*NPG (250 μL; 50 mM), was incubated at appropriate temperature for 10 min. Then 1.5 mL of 1.0 M Na_2_CO_3_ solution was added to terminate the reaction. The amount of *p*-nitrophenol (*p*NP) released was determined spectrophotometrically by reading the absorbance at 405 nm.

The specific activities towards substrates cellobiose and gentiobiose were assayed using the glucose oxidase-peroxidase (GOD-POD) method under the respective optimal conditions. The standard reaction systems consisted of an appropriately diluted enzyme (70 μL) and substrate solution (70 μL) with a concentration of 2 mM in 50 mM pH 4.5 Na_2_HPO_4_-citric acid buffer. After a 10-min incubation at appropriate temperature, the reactions were terminated using a boiling water bath. GOD-POD coloring solution (2.1 mL) was then added into the system, and the absorbances at 520 nm were measured to determine the amount of released glucose. Each experiment was performed in triplicate.

The kinetic parameters, Michaelis constant (*K*_m_) and kinetic constant (*k*_cat_), were determined under the respective optimal conditions of wild-type Bgl3A and all mutants for 5 min in Na_2_HPO_4_-citric acid buffer (100 mM) containing 1–20 mM of different substrates. The data were plotted and fitted by Graphpad 6.0 software (GraphPad Software, San Diego, CA, USA) to calculate the kinetic parameters.

### 2.6. MD Simulation and Calculation of Binding Energy

MD simulations were carried out with the AMBER 18 simulation packages according to the instructions of the reference manual [25,26]. The topologies and parameters of the enzyme and substrate cellobiose in the complex structure were generated by the Amber ff14SB [27] and GLYCAM06-1 force field, respectively. The SHAKE algorithm was employed during the MD simulation process to constrain all bonds relating to hydrogen atoms, and the time step was set as 0.002 ps. The simulation system was immersed in a cubic TIP3P water box with a boundary distance of 1.0 nm for each protein. After, appropriate sodium ions were added to neutralize the negative charge to ensure that the whole system remained electrically neutral. The cutoff radius of the non-bonding interaction was set to 12 Å, and the Particle Mesh Ewald (PME) method was used to deal with the long-range electrostatic interaction. The energy minimization simulation included equilibrium of solvent molecules for 2 ps and minimization of the whole system for 50 ps. The system was slowly heated from 0 K to 300 K under the control of the Langevin algorithm and subsequently equilibrated for 500 ps at 1.0 atmospheric pressure to ensure that the water density reached 1.0 g/cm^3^. Finally, a 100 ns production MD simulation was run under constant pressure, and the coordinate trajectory was recorded every 10 ps. The program, cpptraj, was employed to analyze the generated trajectory files [28].

To calculate the binding free energy, 2000 snapshots with equal intervals were extracted from the 20–100 ns production MD simulation trajectory. Energy analyses were performed using the molecular mechanics/Poisson–Boltzmann surface area (MM/PBSA) method. In this method, the total energy of a solvated molecule could be cataloged into three major items and had the following quantitative relationship Equation (1) [29].

*G* = *E*_bnd_ + *E*_el_ + *E*_vdW_ + *G*_pol_ + *G*_np_ − *TS* (1)

*E*_bnd_, *E*_el_ and *E*_vdW_ represented the standard MM energy terms from bonded (bond, angle, and dihedral), electrostatic and van der Waals interactions. And *G*_pol_ and *G*_np_ are the polar and non-polar contributions to the solvation free energies, respectively. *TS* was the energy component involving the entropy effect. However, Δ*S* was not calculated in this study because the substrates in all complexes were identical and therefore Δ*S* would not significantly impact the results. The difference value between *G*_complex_ and (*G*_receptor_ + *G*_ligand_) was considered the change in Gibbs free energy caused by the binding process, which is approximately equal to the binding energy of the receptor-ligand complex. Thus, the binding free energy (*G*_binding_) was calculated according to the following formula Equation (2):

*G*_binding_ = Δ*E*_el_
*+* Δ*E*_vdW_
*+* Δ*G*_pol_
*+* Δ*G*_np_ (2)

## 3. Results and Discussion

### 3.1. Substrate Binding Analysis

**Figure 1:**
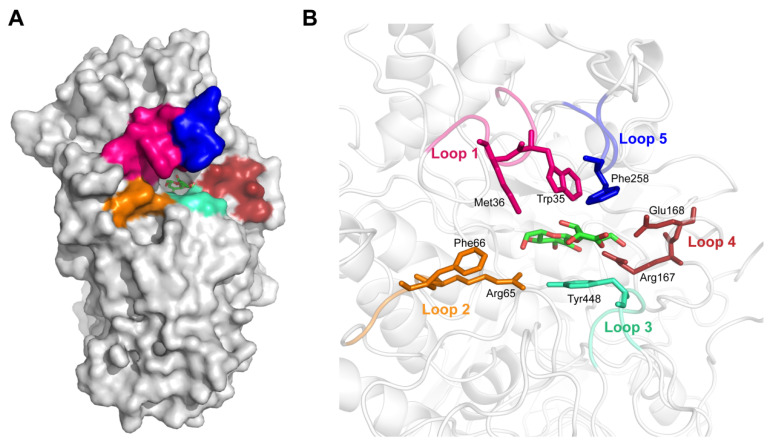
Docking analyses of Bgl3A. Stereoview of modeled Bgl3A and the substrate-binding pocket depicted as surface (**A**) and cartoons and sticks (**B**). Substrate binding sites and five flexible loops surrounding the pocket are indicated.

According to our previous study, poor substrate affinity was the main reason for the low catalytic efficiency of Bgl3A towards natural glycoside substrates. Increasing the enzyme’s substrate affinity was the most direct strategy for improving its catalytic efficiency. To analyze the enzyme’s binding mode, a credible complex structure of Bgl3A and cellobiose was obtained by homologous modeling and molecular docking (evaluation information shown in Supplemental Figure S1). The substrate-binding pocket of Bgl3A was located in a cavity on the inner side of the protein (Figure 1A). Several binding residues were distributed in five flexible loops around the pocket, and they formed interactions with the substrate molecule, resulting in them playing a vital role in catalysis (Figure 1B).

**Figure 2:**
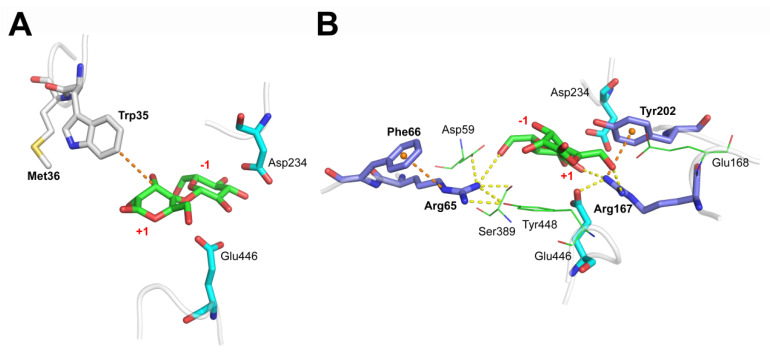
Substrate-binding interactions. Detailed interactions between substrate cellobiose and the hydrophobic Trp35 (**A**) and hydrogen bond-forming Arg65 and Arg167 (**B**). The hydrophobic interaction and the cation-π interactions are shown in dotted brown lines, and the hydrogen bonds are shown in dotted yellow lines.

Two major amino acid groups contributed to the substrate binding. The first group was the hydrophobic region formed by three hydrophobic amino acid residues, Met36, Trp35, and Phe258, which served as the hydrophobic stacking point for the +1 subsite of cellobiose. Among them, the hydrophobic interaction formed between Trp35 and C3/C4 atoms of the substrate +1 subsite was the primary hydrophobic interaction and undertook the role of capturing substrate molecules from the solvent (Figure 2A). Several published structural research studies have drawn a similar conclusion, and demonstrated that Trp68, Phe305, and Tyr511 of *Aa*BGL1 (PDB 4IIB) from *Aspergillus aculeatus* and Trp37, Phe260, and Tyr443 of *Hj*Cel3A (PDB 3ZYZ) from *Hypocrea jecorina* were three conserved residues of great importance for recognizing cellobiose [15,16]. Moreover, the substitution of conserved W Trp48 with Ile at the pocket entrance made HiBgl3B from *Humicola insolens* Y1 to be a strict aryl-β-glucosidase, inhibiting its activity towards all disaccharides [11].

Another group involved in the substrate binding was the polar residues, which formed a hydrogen bond network with the substrate and within themselves. This hydrogen bond network was centered on Arg65 and Arg167 and promoted the formation of the correct substrate conformation and catalytic residues. Further analysis of polar interactions showed that the terminal amine NH_2_ of Arg65 formed a hydrogen bond with the C6-OH in the −1 subsite and interacted broadly with other substrate binding sites such as Asp59, Ser389, and Tyr448. Similarly, Arg167 formed hydrogen bonds with the C6-OH in the +1 subsite, the C2-OH in the −1 subsite, and the OE2 atom of the nucleophilic catalytic residue Glu446 (Figure 2B). The locations and spatial dynamics of these two residues determined the stability and strength of the hydrogen bond network in the binding pocket. Some computational studies also emphasized the importance of these two conserved arginines, which reported that Arg169 and Arg67 stabilized the glucose at the acceptor site (subsite +1). Additionally, disrupting the hydrogen bond networks reduced the affinity and reactivity of a sugar acceptor [30,31].

### 3.2. Mutant Design

**Figure 3:**
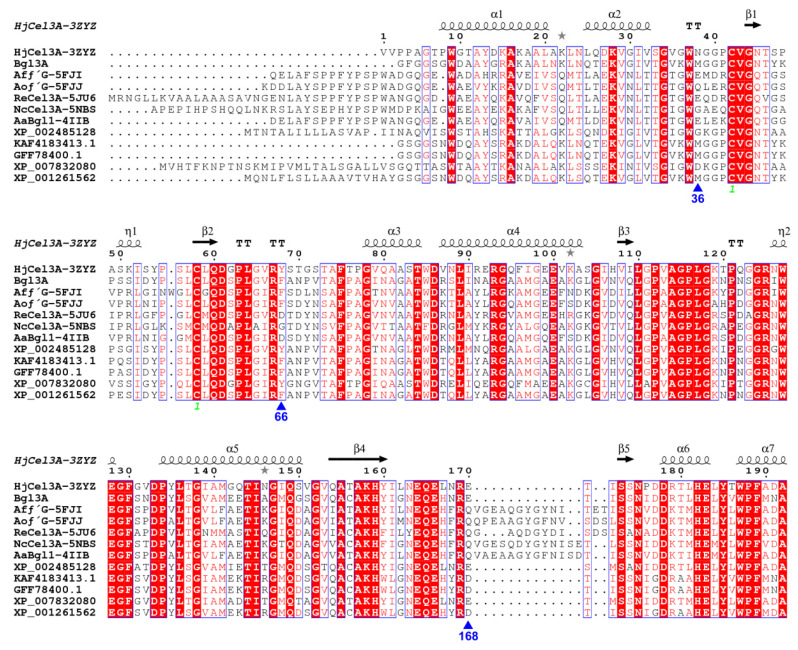
Multiple sequence alignment of Bgl3A with other GH3 β-glucosidases, namely, HjCel3A from *Hypocrea jecorina* (PDB: 3ZYZ) [15], AfβG from *Aspergillus fumigatus* (PDB: 5FJI) and AoβG from *Aspergillus oryzae* (PDB: 5FJJ) [32], ReCel3A from *Rasamsonia emersonii* (PDB: 5JU6) [33], NcCel3A from *Neurospora crassa* OR74A (PDB: 5NBS) [34], AaBgl from *Aspergillus aculeatus* (PDB: 4IIB) [16]. The multiple sequence alignment was performed using ClustalX version 2.1, and rendered by the online tool ESPript (http://espript.ibcp.fr/ESPript/ESPript/). Strictly identical amino acids were showed in red background color and white characters, and groups of similar amino acids were showed in red characters. Blue frames represented similarity across groups.

**Figure 4:**
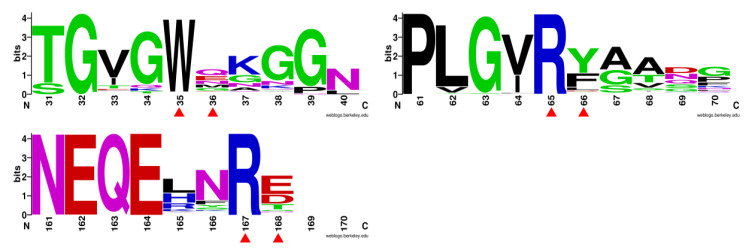
Evolutionary conservation analyses of residues Trp35/Met36, Arg65/Phe66, and Arg167/Glu168. The evolutionary conservation analysis was performed using the Weblogo program (http://weblogo.berkeley.edu/logo.cgi).

Enhancing or stabilizing the binding capacities of these two interaction components was a feasible way to improve the substrate affinity of the enzyme. However, multiple sequence alignment showed that the residues Trp35, Arg65, and Arg167 were almost absolutely conserved in the genetic evolution of GH3 β-glucosidases, which illustrated their importance laterally (Figure 3). As alternatives, three adjacent residues which showed no sequence conservation (Figure 4) were supposed to affect substrate binding by interacting with the previously mentioned three conserved binding sites. We observed that position 36 was close to Trp35 in space, and this might be Gln, Glu, Asp, Asn, and Gly as an alternative to Met in other GH3 β-glucosidases. Therefore, Met36 was chosen as the site-saturation mutagenesis site for catalytic efficiency improvement in Bgl3A. Moreover, aromatic residues Phe66 and Tyr202 were predicted to form pairwise cation-π interactions with the alkaline residues Arg65 and Arg167, respectively. The cation-π interaction is a noncovalent interaction formed between positive cations or groups and benzene rings.

Many studies have documented that the cation-π interaction widely exists in protein structures and enhances the conformational stability of the bonded residues, where Lys or Arg side chains interact with Phe, Tyr, or Trp [35,36]. As with other noncovalent aromatic interactions in protein structure, the cation-π interaction includes a substantial electrostatic component [37] and can be enhanced by increasing the electronegativity of the aromatic rings. Glu168, a negatively charged and genetically variable residue adjacent to Arg167 (Figure 2B), was considered to interfere with the cation-π interaction between Arg65 and Phe66. In summary, site-saturation mutagenesis at position 36 and site-directed mutagenesis of F66Y and E168Q were designed to improve the substrate affinity and catalytic efficiency of the enzyme Bgl3A.

### 3.3. Catalytic Performances of Wild-Type Bgl3A and Its Mutants

Specific activities and kinetic parameters towards *p*NPG and cellobiose of wild-type and all mutant enzymes were determined under respective optimum conditions (75 °C for wild type and most mutant enzymes, and 70 °C for M36V/S/C/K/D). For the artificial substrate *p*NPG, nearly all mutants decreased catalytic efficiencies because of the significant increase in *K*_m_ values (Table 1). This may be because our design targets mainly glycosyl-based substrates. Among mutants at position 36, M36E and M36N exhibited 2.3-fold higher catalytic efficiencies on cellobiose with higher *k*_cat_ values and much lower *K*_m_ values compared with wild-type Bgl3A. Furthermore, F66Y and E168Q also showed 60% and 40% improvement, respectively, in catalytic efficiencies on cellobiose because of the substantially decreased values of *K*_m_, although their *k*_cat_ values were slightly lower than the wild-type (Table 1). The *K*_m_ values of four dominant mutants, M36E (5.2 mM), M36N (4.76 mM), F66Y (4.3 mM), and E168Q (5.0 mM), were approximately halved. This indicated that better substrate affinities with natural glycoside substrates such as cellobiose were obtained by engineering the adjacent residues of conserved binding sites. Further determinations of enzyme performance on gentiobiose confirmed that both mutants M36E and M36N showed certain degrees of improvement in catalytic efficiencies on gentiobiose with lower *K*_m_ values of 2.9 mM and 3.1 mM, respectively, compared with 5.4 mM for wild-type Bgl3A (Table S2).

### 3.4. M36E and M36N Mutations Stabilized Trp35 Conformation by Introducing Hydrogen Bond Interactions

To investigate the mechanism of catalytic efficiency improvement, enzyme-cellobiose complexes of dominant mutants were subjected to a 100 ns MD simulation analysis. The root mean square deviation (RMSD) plots of the α-carbon atoms of five complexes plateaued during the last 80 ns, indicating that the systems maintained equilibrium (Figure S2).

**Figure 5:**
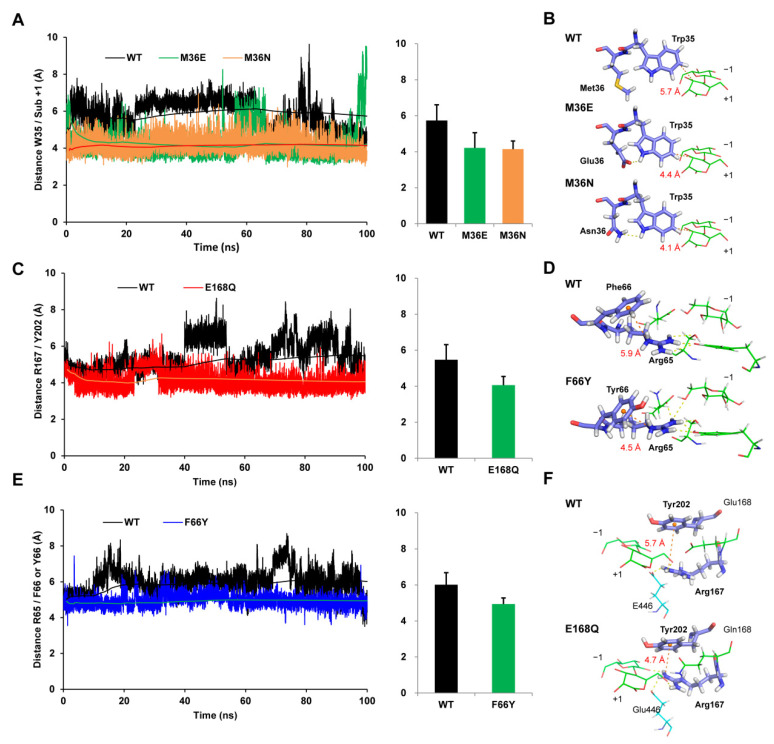
Distances and average conformations of investigated residues during the MD simulations. The distance between atoms Trp35@CH2 and subsite +1@C3 plotted with the simulation time (**A**) and the average conformation (**B**) in the wild-type and mutants (M36E and M36N). The distances between the positively-charged atoms of arginine and the center of mass of the benzene in cation-π interacting residues (Arg65/Phe66 or Tyr 66 and Arg167/Tyr202) plotted along with the simulation time (**C**,**E**) and in the average conformation (**D**,**F**) in the wild-type and mutants F66Y and E168Q, respectively. Dotted yellow lines indicate the hydrogen bonds. The trend of average distance values were shown in lines and the range of distance values were plotted in bar charts.

Stabilizing hydrophobic interactions between the hydrophobic stacking site Trp35 and the C3 atom of subsite +1 was a vitally important factor for substrate binding. Moreover, it is well known that distance was a significant determinant of the strength of hydrophobic interactions [38,39]. As for mutants M36E and M36N, the distances between the atoms Trp35@CH2/subsite +1@C3 were plotted by analyzing the dynamic trajectories and compared with wild-type Bgl3A. It was shown that the atoms Trp35@CH2/subsite +1@C3 departed from each other constantly in the wild-type, which could result in the interruption of the hydrophobic interaction, while mutants M36E and M36N exhibited more stable interaction distances (Figure 5A). Additionally, the distance values of M36E and M36N were measured to be 4.4 Å, and 4.1 Å in the average conformations, respectively, which were much closer than the 5.7 Å observed in wild-type Bgl3A (Figure 5B). Furthermore, statistics of hydrogen bond formation showed that new sets of hydrogen bonds involving the NE1 atom in the imidazole group of Trp35 were introduced in the two mutants, namely, Glu36@OE1/Trp35@NE1 or Glu36@OE2/Trp35@NE1 in M36E with a total occupancy of 38.30 ± 1.34%, and ASN36@ND2/TRP35@NE1 in M36N with an occupancy of 22.46 ± 2.13% (Table 2). These results indicated that substitutions of the neutral and partially hydrophobic residue Met36 with the hydrophilic residue Glu36 or Asn36 gave rise to better stabilities of Trp35 by forming additional hydrogen bonds with the NE1 atom in the imidazole group, and this was in accordance with the changes of root mean square fluctuations (RMSF) of residue Trp35 (Figure S2).

### 3.5. F66Y and E168Q Mutations Enhanced Substrate Binding by Strengthening the Cation-π Interactions

Figure 5C,E showed that the interval distances between the paired residues Arg65/Phe66 and Arg167/Tyr202 varied significantly in the wild-type with an average value of 5.9 Å and 5.7 Å during the simulation, respectively. Correspondingly, the Arg65/Phe66 distance was shortened to 4.5 Å in mutant F66Y, and Arg167/Tyr202 distance was shortened to 4.7 Å in mutant E168Q (Figure 5D,F). Moreover, the migration distances of Arg65 and Arg167 in corresponding mutants decreased significantly compared with those of the wild-type, which could support more stable binding conformations. Compared with the wild-type, the amine atoms of Arg65 in mutant F66Y showed 1.99-fold, 1.90-fold, 1.63-fold, and 6.30-fold hydrogen bond occupancies interacting with Asp59@OD2, Ser389@OG, Tyr448@OH, and Subsite −1@O6, respectively. Additionally, Arg167 in mutant E168Q showed 1.23-fold, 1.68-fold, and 3.36-fold hydrogen bond occupancies interacting with Glu 446@OE2, Subsite +1@O6, and Subsite −1@O2, respectively (Table 2). These results suggested that the cation-π interactions were strengthened in two mutants and promoted the spatial shift of Arg65 and Arg167 toward the positions facilitating the formation of hydrogen bonds with the substrate cellobiose and other polar binding sites (Figure 5D,F). The improved hydrogen bonding abilities then led to better affinities and higher catalytic efficiencies towards cellobiose. However, the increased rigidity of Arg65 and Arg167 may also be responsible for the decrease in *k*_cat_ values.

### 3.6. Binding Free Energy Calculation

The binding free energies calculated by MM/PBSA method are shown in Table 3. It is worth noting that the lower binding free energy represented a stronger substrate affinity [40]. The mutants M36E, M36N, F66Y, and E168Q had lower binding free energies compared with the wild-type (−22.50 kcal/mol), indicating that better combinations with cellobiose were successfully achieved. Moreover, the calculated substrate affinities were ranked in the order of F66Y > M36N ≥ M36E > E168Q > WT, which was in agreement with the experimentally determined *K*_m_ values. The change value of the non-polar component of solvation free energy Δ*E*_np_ of mutants M36N and M36E were −7.46 kcal/mol and −9.06 kcal/mol, respectively were lower than that of the wild-type and probably contributed to the improved hydrophobic interaction between the stacking site Trp35 and subsite +1. Furthermore, the polar energy components, Δ*E*_el_ and Δ*E*_p__ol_ of the mutants F66Y and E168Q, were significantly lower than the wild-type and contributed the primary positive effect in reducing total binding free energies. Therefore, the improvement of substrate affinities of mutants F66Y and E168Q was achievable by strengthening the hydrogen bond networks centered on Arg65 and Arg167.

It is worth mentioning that the whole catalytic cycle includes three equally important processes: substrate binding, reaction, and product release. Generally, mutations in key sites of the enzyme activity center might affect all three processes simultaneously, which is finally reflected in the change of enzymatic reaction kinetic parameters. The *K*_m_ values measured experimentally were the apparent binding constants reflecting the substrate concentration requirement for the whole catalytic process of enzymes. In this study, the *K*_m_ values of the dominant mutants were significantly reduced, indicating that the substrate concentrations required by the enzymes to exert normal catalytic activities were reduced. This was direct evidence of the enzyme’s improved ability to bind substrates. Besides, MD simulation and MMPBSA analysis showed that the binding energy of the mutants to the substrate was enhanced, which was consistent with the experimental results. Respect to the product, there was reason to believe that the enhanced +1 binding ability could lead to slowdown in product release. However, the distance between the two glucose molecules generated by the hydrolysis of the substrate will be larger than the distance between the two glycosylated glucosyl units before the reaction due to the break of glycosidic bonds. This meant that the +1 position of the released glucose molecule is forced to move toward the outside of the catalytic pocket, resulting in changes in binding interactions with the enzyme molecule, such as partial hydrogen bond breaking or weakening. Therefore, it could be inferred that the improvement of binding ability at +1 sites has a greater impact on substrate binding than on product release, which was beneficial to improve enzyme catalytic efficiency.

## 4. Conclusions

Three conserved residues (Trp36, Arg65, and Arg167) around the subsite +1 were suspected to be directly involved in the binding of glycoside substrates. Correspondingly, three adjacent residues (Met36, Phe66, and Gln168) showing variability in genetic evolution were supposed to significantly affect the conformation of the aforementioned conserved residues, therefore influencing the substrates binding. In this study, the substrate affinity and catalytic efficiency of Bgl3A were successfully improved to different degrees by engineering three residues adjacent to the conserved binding sites and indirectly taking effect, which properly conformed to our design. Consequently, the dominant mutants exhibited higher affinities and 1.4–2.3-fold catalytic efficiencies towards cellobiose. Structural and MD simulation analyses suggested that binding free energies of GH3 β-glucosidases could be obtained by either strengthened hydrophobic interactions between stacking aromatic residues and the substrate or stabilized hydrogen-bonding networks in the binding pocket. This work is expected to broaden the understanding of the substrate combination of GH3 β-glucosidases.

## Figures and Tables

**Table 1:** Reaction optimum, specific activities, and kinetic parameters of wild-type Bgl3A and its mutants towards *p*NPG and cellobiose.

Enzymes	Optima		*p*NPG				Cellobiose				Fold Change in *k*_cat_/*K*_m_ on Cellobiose	Activity Ratio _Cellobiose/*p*NPG_
	T (°C)	pH	Specific Actvitity (U·mg^–1^)	*K*_m_ (mM)	*k*_cat_ (s^–1^)	*k*_cat_/*K*_m_ (s^–1^·mM^–1^)	Specific Actvitity (U·mg^–1^)	*K*_m_ (mM)	*k*_cat_ (s^–1^)	*k*_cat_/*K*_m_ (s^–1^·mM^–1^)		
WT	75	4.5	905.0 ± 11.3	0.18 ± 0.03	1664.3 ± 26.9	9096.0	265.5 ± 3.9	10.4 ± 0.4	786.0 ± 9.6	75.8	1.0	0.29
F66Y	75	4.5	892.9 ± 19.6	0.24 ± 0.02	1773.3 ± 11.9	7451.0	341.0 ± 4.7	4.3 ± 0.3	511.7 ± 11.2	119.0	1.6	0.38
E168Q	75	4.5	858.9 ± 12.4	0.21 ± 0.03	1744.2 ± 27.8	8385.6	236.1 ± 6.8	5.2 ± 0.2	540.6 ± 8.6	104.0	1.4	0.27
M36E	75	4.5	766.0 ± 21.2	0.25 ± 0.03	1798.0 ± 31.6	7088.0	256.6 ± 4.6	5.0 ± 0.7	875.4 ± 6.9	174.2	2.3	0.33
M36N	75	4.5	850.8 ± 17.1	0.59 ± 0.07	1067.3 ± 33.7	1794.1	247.9 ± 3.7	4.8 ± 0.4	843.5 ± 7.1	177.2	2.3	0.29
M36G	75	4.5	907.8 ± 12.1	1.10 ± 0.06	2566.4 ± 15.9	2320.0	75.2 ± 2.8	5.4 ± 1.3	115.0 ± 3.1	21.5	0.3	0.08
M36A	75	4.5	876.9 ± 26.8	1.40 ± 0.13	1561.1 ± 16.8	1080.9	29.4 ± 1.2	5.3 ± 0.7	70.8 ± 2.7	13.3	0.2	0.03
M36V	70	4.5	478.4 ± 11.7	0.80 ± 0.04	817.8 ± 21.6	1038.6	25.8 ± 1.6	5.2 ± 1.2	54.4 ± 1.6	10.5	0.1	0.05
M36L	75	4.5	483.6 ± 15.4	0.66 ± 0.12	1279.1 ± 14.8	1932.4	54.5 ± 1.7	3.9 ± 0.2	89.9 ± 3.9	23.2	0.3	0.11
M36I	75	4.5	390.2 ± 11.8	1.24 ± 0.09	1304.5 ± 29.7	1053.2	34.9 ± 1.2	4.8 ± 0.5	88.8 ± 3.7	18.4	0.2	0.09
M36P	75	4.5	23.9 ± 3.4	2.10 ± 0.13	195.5 ± 11.2	93.0	2.9 ± 0.3	25.1 ± 1.4	22.5 ± 1.4	0.9	0.0	0.12
M36S	70	4.5	32.8 ± 2.7	2.26 ± 0.27	105.8 ± 7.9	46.8	2.4 ± 0.5	5.7 ± 0.4	5.6 ± 0.6	1.0	0.0	0.07
M36C	70	4.5	614.6 ± 9.7	0.97 ± 0.15	1888.5 ± 51.3	1956.9	57.8 ± 2.9	4.1 ± 0.7	116.9 ± 6.4	28.9	0.4	0.09
M36T	75	4.5	593.2 ± 13.8	0.75 ± 0.07	1688.0 ± 15.9	2250.7	63.6 ± 3.7	5.7 ± 1.1	185.5 ± 5.1	32.7	0.4	0.11
M36Q	75	4.5	408.6 ± 10.6	0.83 ± 0.13	860.4 ± 21.8	1039.8	27.4 ± 0.8	3.0 ± 0.2	41.0 ± 2.8	13.9	0.2	0.07
M36W	75	4.5	212.6 ± 8.7	1.23 ± 0.22	1701.8 ± 15.7	1386.2	63.8 ± 0.9	7.3 ± 0.2	83.4 ± 3.4	11.4	0.2	0.30
M36Y	75	4.5	348.6 ± 16.9	1.84 ± 0.18	2684.3 ± 19.4	1458.9	35.8 ± 1.3	6.1 ± 0.1	48.5 ± 1.8	7.9	0.1	0.10
M36F	75	4.5	170.9 ± 3.7	1.67 ± 0.17	918.9 ± 21.9	551.3	24.3 ± 0.6	6.7 ± 0.3	48.5 ± 3.1	7.3	0.1	0.14
M36R	70	4.5	589.1 ± 16.9	0.99 ± 0.11	1110.0 ± 11.7	1114.7	21.8 ± 1.1	2.5 ± 0.2	37.6 ± 1.1	14.9	0.2	0.04
M36H	75	4.5	194.5 ± 21.3	1.24 ± 0.18	1382.1 ± 10.8	1114.6	27.8 ± 1.1	29.0 ± 3.7	737.8 ± 21.3	25.1	0.3	0.14
M36K	70	4.5	208.9 ± 19.7	1.18 ± 0.12	1854.5 ± 26.9	1571.6	25.9 ± 0.9	27.1 ± 2.9	730.7 ± 33.7	26.7	0.4	0.12
M36D	70	4.5	93.5 ± 8.6	1.94 ± 0.07	633.2 ± 9.1	325.8	16.5 ± 1.7	6.8 ± 0.7	31.1 ± 2.7	4.6	0.1	0.18

**Table 2:** Hydrogen bond occupancy (%) during the last 80 ns of the MD simulations.

Acceptor	Donor H	Donor	Hydrogen Bond Occupancy (%) ^a^				
			WT	M36E	M36N	F66Y	E168Q
Trp35							
Asn36@ND2	Trp35@HE1	Trp35@NE1	- ^b^	-	22.46 ± 2.13%	-	-
Glu36@OE1	Trp35@HE1	Trp35@NE1	-	38.30 ± 1.34%	-	-	-
Arg65							
Asp59@OD2	Arg65@HH11	Arg65@NH1	35.93 ± 2.37%	39.34 ± 1.47%	32.46 ± 1.68%	71.48 ± 5.16%	27.67 ± 0.94%
Subsite −1@O6	Arg65@HH11	Arg65@NH1	7.09 ± 2.11%	13.93 ± 2.13%	10.14 ± 1.59%	44.64 ± 3.45%	8.11 ± 1.12%
Ser389@OG	Arg65@HH12	Arg65@NH1	45.20 ± 4.69%	47.08 ± 3.18%	39.74 ± 2.26%	86.03 ± 5.34%	43.61 ± 3.17%
Tyr448@OH	Arg65@HH12	Arg65@NH1	79.88 ± 3.97%	74.71 ± 2.26%	79.16 ± 4.13%	97.85 ± 3.89%	86.82 ± 5.33%
Tyr448@OH	Arg65@HH22	Arg65@NH2	12.86 ± 1.26%	19.25 ± 3.14%	18.95 ± 2.33%	53.11 ± 4.11%	15.14 ± 2.37%
Arg167							
Glu 446@OE2	Arg167@HH22	Arg167@NH2	77.88 ± 1.98%	79.84 ± 6.13%	84.84 ± 3.11%	73.48 ± 2.37%	96.13 ± 3.51%
Subsite +1@O6	Arg167@HH12	Arg167@NH1	29.31 ± 1.31%	34.44 ± 3.17%	30.67 ± 3.21%	23.25 ± 2.15%	49.32 ± 4.11%
Subsite −1@O2	Arg167@HH22	Arg167@NH2	11.17 ± 2.18%	13.62 ± 2.31%	8.26 ± 0.91%	15.03 ± 1.13%	37.53 ± 2.34%

^a^ The occupancy was the proportion of frames forming hydrogen bonds in the total frames during the MD simulation. A geometric consideration (distance of 3.5 Å and an angle cutoff of 135°) was used as the criteria for hydrogen bonding. ^b^ No hydrogen bond was formed between the selected residues.

**Table 3:** Calculated energy components and binding free energies (kcal/mol) of cellobiose complexes of wild-type Bgl3A and the mutants M36E, M36N, F66Y, and E168Q.

Energy (kcal/mol)	WT	M36E	M36N	F66Y	E168Q
Δ*E*_vdW_	−34.84 ± 0.39	−36.48 ± 0.70	−38.17 ± 0.49	−34.72 ± 0.99	−34.29 ± 0.78
Δ*E*_el_	−85.13 ± 0.53	−83.79 ± 1.34	−83.67 ± 0.67	−89.11 ± 0.88	−90.22 ± 1.16
Δ*E*_pol_	103.07 ± 1.75	100.21 ± 1.21	100.77 ± 1.12	97.69 ± 0.92	99.40 ± 0.83
Δ*E*_np_	−5.60 ± 0.11	−7.46 ± 0.21	−9.06 ± 0.40	−7.10 ± 0.30	−5.94 ± 0.51
*G*_binding_	−22.50 ± 0.72	−27.52 ± 0.88	−30.13 ± 0.78	−33.25 ± 1.08	−31.05 ± 0.69

## Data Availability

Data is contained within the article or Supplementary Materials.

## Supplementary Materials

The following are available online at https://www.mdpi.com/article/10.3390/biom11121882/s1, Table S1: Primers used in this study, Table S2: Specific activities and kinetic parameters of wild type Bgl3A and its mutants on cellobiose and gentiobiose, Figure S1: Structural evaluation of modeled Bgl3A by PROCHECK and Verify3D programs, Figure S2: Root mean square deviation (RMSD) and root mean square fluctuations (RMSF) plots.
