# Supplementary material for: Improving the Substrate Affinity and Catalytic Efficiency of β-Glucosidase Bgl3A from Talaromyces leycettanus JCM12802 by Rational Design
Source: Biomolecules. 2021 Dec 15;11(12):1882. doi: 10.3390/biom11121882 (PMC8699594; doi:10.3390/biom11121882)
Supplement: Supplementary file 1 [file biomolecules-11-01882-s001.zip › biomolecules-1449461-supplementary.pdf]

Supplementary Material

Improving the substrate affinity and catalytic efficiency of β-glucosidase Bgl3A from *Talaromyces leycettanus* JCM12802 by rational design

Wei Xia[3], Yingguo Bai[2], Pengjun Shi[1*]

[1] Institute of Food Science and Technology, Chinese Academy of Agricultural Sciences, Beijing, China.

[2] Institute of Animal Science, Chinese Academy of Agricultural Sciences, Beijing 100193, PR China

[3] State Key Laboratory of Food Science and Technology, Jiangnan University, 1800 Lihu Avenue, Wuxi, 214122, China

[*]Corresponding author

Pengjun Shi

Institute of Food Science and Technology, Chinese Academy of Agricultural Sciences, NO.2 Yuanmingyuan West Road, Haidian District, Beijing, 100081, People's Republic of China.

E-mail: shipengjun@caas.cn.

Table S1. Primers used in this study.

Primer name	Primer sequence (5′→3′) [a]
Bgl3A-PF	GGGGAATTCTATGGCTTCGGCGGCTCTGGCTG
Bgl3A-PR	GGGGCGGCCGCTCAAATACGGAAAGATTCCTGCT
M36 Saturation -F	ACCGGTGTCAAGTGGNNKGGCGGCCCTTGTGTT
M36 Saturation -R	AACACAAGGGCCGCCMNNCCACTTGACACCGGT
E168Q-F	AGCAAGAGCACAACCGTCAGACCATCAGCTCCAACAT
E168Q-R	ATGTTGGAGCTGATGGTCTGACGGTTGTGCTCTTGCT
F66Y-F	TCCTCTCGGGGTGCGTACTGCCAACCCTGTGACTGCC
F66Y-R	GGCAGTCACAGGGTTGGCGTAACGCACCCCGAGAGGA

[a] The restriction sites and mutation codons are shown underlined.

Table S2. Specific activities and kinetic parameters of wild type Bgl3A and its mutants on cellobiose and gentiobiose.

Substrate	Enzyme	Specific actvitity (U/mg)	K_m (mM)	k_{cat} (/s)	k_{cat}/K_m (/s/mM)	Fold change
Gentiobiose	WT	393.2	5.4	877.6	164.1	1.00
	M36E	384.5	2.9	716.5	245.3	1.5
	M36N	328.4	3.1	611.7	194.7	1.2

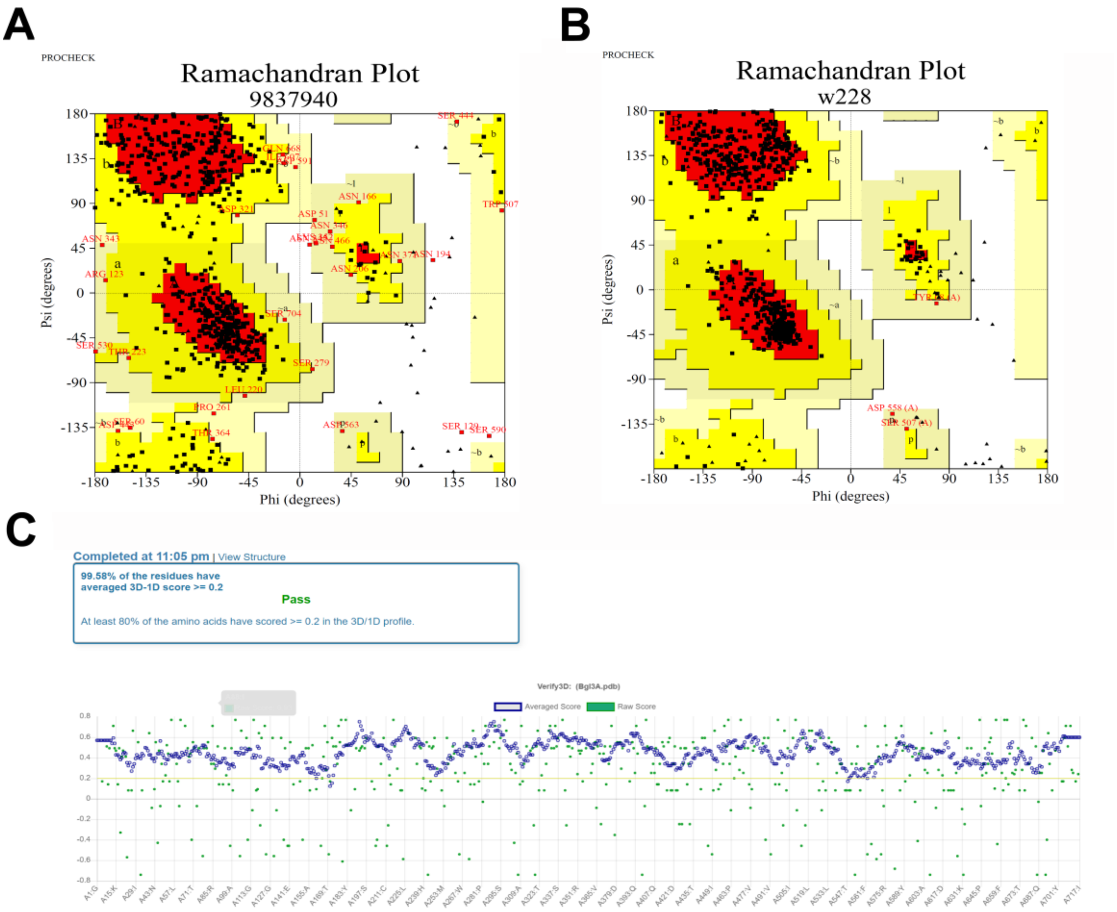

Figure S1. Structural evaluation of modeled Bgl3A by PROCHECK and Verify3D programs. Results showed that (A) 68.7% and 26.6% residues were in the core and allowed regions of the Ramachandran plot, and (C) 99.58% of residues have averaged 3D-1D score ⩾ 0.2 in the original modeled structure of Bgl3A. And 91.0% and 8.5% residues were in the core and allowed regions of the Ramachandran plot in the energy minimized structure of Bgl3A.

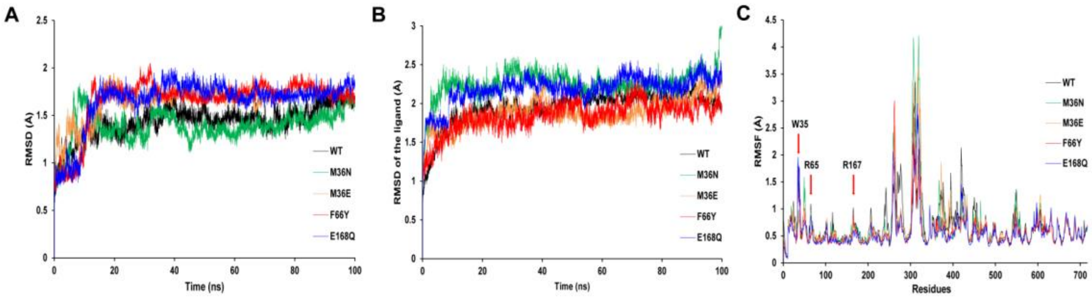

Figure S2. Root mean square deviation (RMSD) plots of the protein backbone (A) and the substrate (B) from the starting position, and root mean square fluctuations (RMSF) plots (C) of the five complexes during the MD simulation.
